# Draft Genome Sequence of C:P1.5-1,10-8:F3-6:ST-11 Meningococcal Clinical Isolate Associated with a Cluster on a Cruise Ship

**DOI:** 10.1128/genomeA.01263-14

**Published:** 2014-12-04

**Authors:** A. Neri, C. Fazio, A. Ciammaruconi, A. Anselmo, A. Fortunato, A. Palozzi, P. Vacca, S. Fillo, F. Lista, P. Stefanelli

**Affiliations:** a Department of Infectious, Parasitic & Immuno-mediated Diseases, Istituto Superiore di Sanità, Rome, Italy; b Army Medical and Veterinary Research Center, Rome, Italy

## Abstract

Meningococcal serogroup C strains, in particular those belonging to the ST-11 clonal complex, are known to cause invasive diseases worldwide. We report the genome sequence of a *Neisseria meningitidis* strain linked to a cluster of cases of invasive meningococcal disease on a cruise ship that was described in 2012.

## GENOME ANNOUNCEMENT

Meningococcal serogroup C strains belonging to the ST-11 clonal complex (cc) are known to cause invasive diseases worldwide (1, 2). Several reports indicate an increase of severe cases and outbreaks of invasive diseases due to serogroup C meningococci belonging to ST-11 cc, characterized by a high mortality rate (3–5). A cluster of four cases of invasive meningococcal disease due to *N. meningitidis* of serogroup C ST-11 cc that occurred on a cruise ship in October 2012 was reported in Italy (6).

Here, the draft genome sequence of one of the four *N. meningitidis* strains, C:P1.5-1,10-8:F3-6:ST-11, isolated from a patient who died, is described.

Genomic DNA was extracted using QIAamp DNA minikit (Qiagen, Hilden, Germany) from an overnight culture grown on Thayer Martin agar. Whole-genome sequencing was performed using Illumina MiSeq sequencer, which generated 2,023,619 total reads (1,752,365 after trimming) with an average length of 180 × 2  nucleotides (nt) (~613 Mb). A second sequencing run was performed using a whole-genome shotgun strategy with a Roche genome sequencer (GS) FLX 454 FLX+ (Roche, Basel, Switzerland). In this second sequencing run, 71,631 reads were generated, with an average length of 550 nt (~40 Mb). The final coverage was 297×. *De novo* assemblies of Illumina and Roche reads were performed by ABySS software version 1.3.5 (7) and GS De Novo Assembler version 2.8 (454 Life Science, Branford, CT, USA), respectively. The resulting contigs from both sequencings were merged when overlapping using Minimus2 software (8): the final number of contigs was 102, and the draft genome sequence was 2,190,379 bp long.

The 102 contigs were submitted to the NCBI whole-genome shotgun (WGS) submission portal (https://submit.ncbi.nlm.nih.gov/subs/wgs/), and the sequence was annotated using the NCBI Prokaryotic Genome Automatic Annotation Pipeline (PGAAP) (http://www.ncbi.nlm.nih.gov/genome/annotation_prok/).

### Nucleotide sequence accession numbers.

This whole-genome shotgun project has been deposited at DDBJ/EMBL/GenBank under the accession no. JPTA00000000. The version described in this paper is version JPTA01000000.
